# Sophocarpine Alleviates Injury-Induced Intima Hyperplasia of Carotid Arteries by Suppressing Inflammation in a Rat Model

**DOI:** 10.3390/jcm10225449

**Published:** 2021-11-22

**Authors:** Genhuan Yang, Rong Zeng, Xitao Song, Changwei Liu, Leng Ni

**Affiliations:** 1 Department of Vascular Surgery, Beijing Tiantan Hospital, Capital Medical University, Beijing 100070, China; yanggenhuan@bjtth.org; 2 Department of Vascular Surgery, Peking Union Medical College Hospital, Peking Union Medical College and Chinese Academy of Medical Sciences, Beijing 100730, China; zengrong@pumch.cn (R.Z.); songxitao@pumch.cn (X.S.); liucw@pumch.cn (C.L.)

**Keywords:** sophocarpine, balloon injury, vascular, intima hyperplasia, angioplasty

## Abstract

Introduction: Balloon angioplasty is a commonly applied procedure for treating atherosclerotic vascular diseases. However, the maintenance of long-term lumen patency is relatively difficult due to the occurrence of restenosis. Previous research has shown that the occurrence of vascular wall inflammation is associated with higher rates of restenosis. Sophocarpine (SPC) can exert various therapeutic effects such as anti-oxidation, anti-inflammation, anti-tumor, antivirus and immune regulation. This study aimed to investigate whether SPC can alleviate intimal hyperplasia following balloon injury in a rat carotid artery model. Methods: Twenty Sprague–Dawley rats were randomly assigned to four groups: (i) control, (ii) balloon injury, (iii) balloon injury followed by saline injection, and (iv) balloon injury followed by SPC administration. Each group contained five rats. A high-pressure balloon of 3 mm × 20 mm was placed in the carotid artery. The balloon was inflated to a pressure of 8 atmospheres to carry out rat carotid artery balloon injury model. The areas of neointimal and media were determined by Verhoeff_Van Gieson staining, and the intima-to-media (I:M) ratios were subsequently evaluated. After that, the protein levels of IL-6, IL-1β, MCP-1, NF-κB, TNF-α, VCAM-1, ICAM-1 and eNOS were measured. Results: The ratio of I:M was remarkably higher in the balloon injury group than in the control group (*p* < 0.01). SPC could significantly decrease the ratio of I:M compared with the balloon injury group (*p* < 0.01). Besides, the protein levels of IL-6, IL-1β, MCP-1, NF-κB, TNF-α, ICAM-1 and VCAM-1 were increased in rat carotid arteries exposed to balloon injury (*p* < 0.01), and treatment with SPC could attenuate these effects (*p* < 0.05). Furthermore, balloon injury inhibited the protein expression of eNOS (*p* < 0.01), and SPC could elevate its level (*p* < 0.05). Conclusions: SPC could alleviate an intimal hyperplasia in balloon-injured carotid artery, and the mechanisms underlying this protective effect might be due to its inhibitory potency against inflammation signals. Our study also implies the potential applicability of SPC in treating restenosis after balloon angioplasty.

## 1. Introduction

As we all known, cardiovascular diseases, currently thought to account for about one third of global deaths, rank first among all the diseases that cause human death [1]. Atherosclerosis is one of the most prominent risk factors for cardiovascular diseases, and can result in the formation of atherosclerotic plaque and stenosis of vascular lumen [2]. The most common cardiovascular diseases include peripheral arterial diseases, stroke, and myocardial infarction. Increasing evidence has revealed that the pathogenesis of atherosclerosis involves an inflammatory reaction in the vascular wall via inflammatory mediator production and inflammatory cell infiltration [3]. In clinic, balloon angioplasty, a minimally invasive procedure, has been commonly applied for treating atherosclerotic vascular diseases. However, the maintenance of long-term lumen patency is relatively difficult due to the occurrence of restenosis [4], and the main pathological change involved in vascular restenosis is intimal hyperplasia [5]. Previous research has shown that the occurrence of vascular wall inflammation is associated with a higher rate of restenosis [6]. More notably, the serum of cardiovascular disease patients contains a large quantity of inflammatory mediators, including interleukin-6 (IL-6), interleukin-1β (IL-1β), C-reactive protein, and tumor necrosis factor-α (TNF-α) [7]. Thus, it is essential to develop a novel therapeutic agent for reducing inflammation and preventing restenosis in cardiovascular disease patients treated with angioplasty.

Sophora flavescens is a traditional Chinese medicine that has often been applied to cure a number of diseases since ancient times. As a typical sophora alkaloid, sophocarpine (SPC) is extracted from Sophora flavescens [8]. Sophora alkaloids have lots of common structural features and possess beneficial properties such as immune regulation, anti-tumor, antivirus, anti-inflammation, and anti-oxidation [9]. Sophora alopecuroides Linn. (Kudouzi) is most commonly used in curing fever, edema, inflammation, pain, and so on [10]. Studies have revealed that SPC can suppress the expression of TNF-α and IL-6 in murine macrophage cells and alleviate cachectic symptoms in mice with colon-26 adenocarcinoma [11]. Besides, SPC can reduce the activity of myeloperoxidase, infiltration of neutrophil, and production of inflammatory mediators [12]. Moreover, SPC administration has become an effective way to cure viral myocarditis in clinical trials [10]. In view of the above studies, it is hypothesized that SPC may exert protective effects against balloon angioplasty-induced vascular injury.

In this research, we investigated the protective effects of SPC on the formation of neointimal hyperplasia and expression of inflammatory mediators in a rat carotid artery balloon injury model. To our knowledge, this is the first study to examine the possible roles of SPC in mediating carotid artery stenosis induced by balloon angioplasty.

## 2. Materials and Methods

### 2.1. Animals

The animal care and experimental procedures used in this study were in accordance with those of the National Institutes of Health (the 8th Edition, NRC 2011), and were approved by the Animal Care Committee of Peking Union Medical College. Twenty Sprague-Dawley rats (male; mean body weight = 350 g; 2 months old) were obtained and randomly assigned to 4 different groups (*n* = 5 per group) by random number method: (i) balloon injury group, (ii) control group, (iii) balloon injury plus saline injection group, and (iv) balloon injury plus SPC administration group. A well-established rat carotid artery balloon injury model was conducted as described in a previous study on the principle of blindness [13], except that we used a high-pressure balloon of 3 mm × 20 mm (Boston Scientific, Natick, MA, USA). The balloon pressure was inflated to 8 atmospheres for 30 s. And the animals in balloon injury plus SPC administration group were treated daily with 40 mg/kg of SPC (Sigma-Aldrich, St. Louis, MO, USA) via intraperitoneal injection [14]; while those in balloon injury plus saline injection group received an equivalent volume of saline solution. After 14 days of balloon-induced injury, the rats were anesthetized with a single intraperitoneal injection of 30 mg/kg pentobarbital sodium (Sunbiotech, Beijing, China). Then, excision of the injured carotid arteries was carried out. Finally, a half-length of each carotid artery was stored in 4% paraformaldehyde, while the remaining half was stored in liquid nitrogen.

### 2.2. Histological Analysis

The collected carotid arteries were dehydrated through a graded series of alcohol, transferred to xylene, embedded in paraffin, and serially sectioned at a thickness of 4 μm. Then, all sections were stained with Verhoeff–Van Gieson using an Elastic Stain Kit (Sigma-Aldrich, St. Louis, MO, USA) according to the manufacturer’s instructions. The stained sections were examined and photographed under a Leica DMI 4000 B microscope (Leica Microsystems Ltd. Wetzlar, Germany) at a magnification of 100×. Finally, the images were quantitatively analyzed by an Image-Pro Plus software (ver. 6.0; Media Cybernetics Inc., Bethesda, MD, USA, 2010) on the principle of blindness, and the intima-to-media (I:M) ratios were calculated.

### 2.3. Western Blot Analysis

The frozen balloon-injured arteries were mixed with 600 μL lysis buffer (62.5 mM Tris-HCl, 10% glycerol, and 2% SDS; pH 6.8), and then homogenized using a mortar. The cell lysates were then heated at 100 °C for 10 min, followed by centrifugation at 10,800× *g* for 15 min. The amount of protein in the lysates was determined with a BCA Assay (Thermo Fisher Scientific Inc., Waltham, MA, USA). Equal volumes (20 μg) of extracted proteins were separated through 12% SDS-PAGE (Sunshine Biotechnology Co., Ltd., Nanjing, Jiangsu, China), and subsequently transferred onto polyvinylidene fluoride (PVDF) membranes (Merck Millipore, Billerica, MA, USA). The membranes were then blocked with 5% skimmed milk (Applygen Tech Inc., Beijing, China) in TBST (137 mM NaCl, 20 mM Tris-HCl and 0.05% Tween-20) at room temperature for 1 h. After rinsing three times in TBST, the blocked membranes were incubated overnight at 4 °C with the following primary antibodies: IL-6 (1:50; Boster Biological Tech Ltd., Wuhan, Hubei, China), IL-1β (1:50; Boster Biological Tech Ltd., Wuhan, Hubei, China), MCP-1 (1:200; Santa Cruz Biotechnology Inc., Texas, CA, USA), NF-κB (1:200; Cell Signaling Tech Inc., Boston, MA, USA), TNF-α (1:50; Boster Biological Tech Ltd., Wuhan, Hubei, China), VCAM-1 (1:50; Boster Biological Tech Ltd., Wuhan, Hubei, China), and eNOS (1:200; Cell Signaling Technology Inc., Boston, MA, USA), and ICAM-1 (1:50; Boster Biological Tech Ltd., Wuhan, Hubei, China). After incubation, the membranes were rinsed 3 times with TBST and then incubated with the corresponding secondary antibodies (1:2500; Zhongshan Jinqiao Biotechnology, Beijing, China) at room temperature for 1 h. Lastly, the resulting bands were visualized using an enhanced chemiluminescent reagent kit (TransGen Biotech, Beijing, China). The relative grayscale values of the target proteins were determined with AlphaEase FC software (ver. 4.0; Alpha Innotech Corp., San Leandro, CA, USA, 2012) on the principle of blindness.

### 2.4. Statistical Analysis

All statistical analyses were conducted using SPSS software (ver. 17.0; SPSS Inc., Chicago, IL, USA, 2008), and the data were presented as mean ± standard deviation (SD). One-way ANOVA was employed to compare the means of two groups. A *p*-value of ≤0.05 was deemed statistically significant.

## 3. Results

### 3.1. Balloon Injury Induces Stenosis in Rat Carotid Artery, and this Process Can Be Prevented by SPC

**Figure 1:**
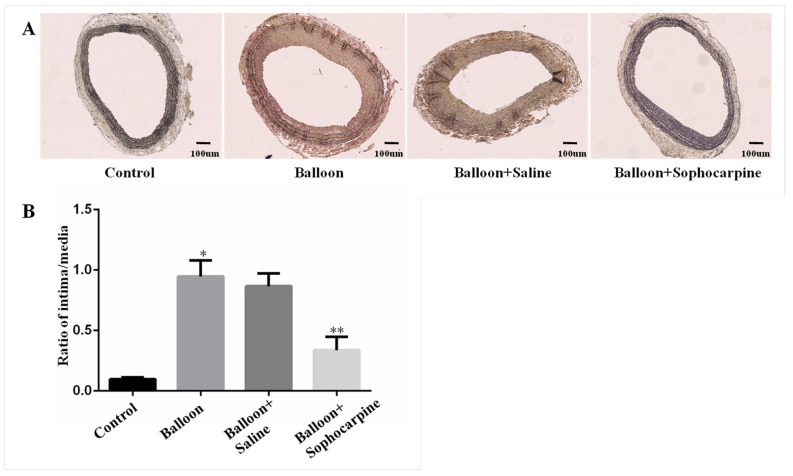
Degree of stenosis in carotid arteries treated differently. (**A**) Verhoeff–Van Gieson stained sections of carotid arteries from the rats in various groups (×100). (**B**) The intima-to-media ration of carotid arteries from the rats in various groups. * *p* < 0.01 versus the control group, ** *p* < 0.01 versus the balloon group.

Histological analysis showed that an intimal hyperplasia was triggered by balloon injury in rat carotid arteries. However, the process of intimal hyperplasia could be attenuated by SPC. Representative cross-sectional areas of the carotid intima and media of the four groups are displayed in Figure 1A. The I:M area was used to measure the degree of lumen stenosis. As shown in Figure 1B, the ratio of I:M was remarkably decreased in balloon injury plus SPC administration group when compared to balloon injury group and balloon injury plus saline injection group (*p* < 0.01). These findings reveal that balloon injury induces luminal stenosis in carotid arteries, and this process can be effectively prevented by SPC.

### 3.2. Balloon Injury Increases the Protein Levels of IL-6, IL-1β, MCP-1, NF-κB, TNF-α, ICAM-1 and VCAM-1, and These Effects Can Be Alleviated by SPC

**Figure 2:**
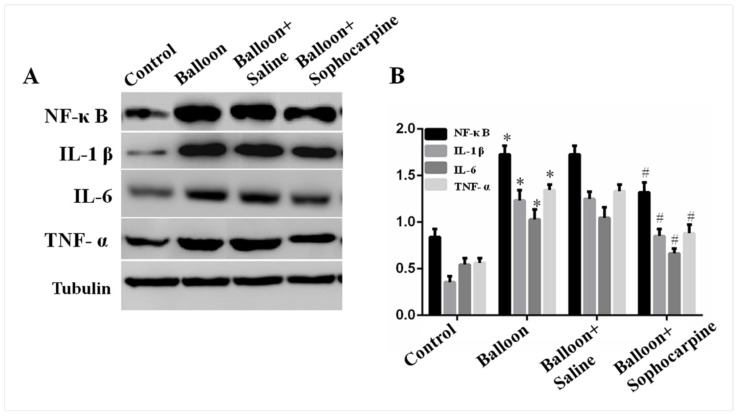
Expression of NF-κB, IL-1β, IL-6 and TNF-α in various groups. (**A**) Western blot analysis showed that balloon injury significantly induced the expression of NF-κB, IL-1β, IL-6 and TNF-α, while sophocarpine decreased the expression. (**B**) Quantification of the Western blot results. * *p* < 0.01 versus the control group, # *p* < 0.05 versus the balloon group.

**Figure 3:**
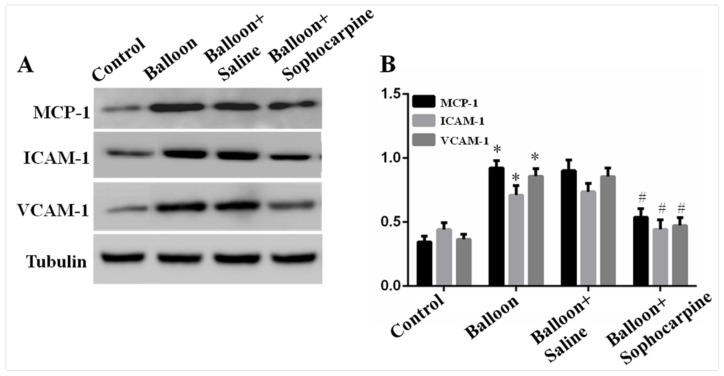
Expression of MCP-1, VCAM-1 and ICAM-1 in various groups. (**A**) Western blot analysis showed that balloon injury significantly induced the expression of MCP-1, VCAM-1 and ICAM-1, while sophocarpine depressed the expression. (**B**) Quantification of the Western blot results. * *p* < 0.01 versus the control group, # *p* < 0.05 versus the balloon group.

The protein levels of IL-6, IL-1β, MCP-1, NF-κB, TNF-α, ICAM-1 and VCAM-1 were assessed by Western blotting. Notably, the protein levels of IL-6, IL-1β, MCP-1, NF-κB, TNF-α, ICAM-1 and VCAM-1 were obviously higher in balloon injury group (*p* < 0.01) than those in control group (Figure 2 and Figure 3). More importantly, SPC markedly decreased the expression levels (*p* < 0.05) of the target proteins (Figure 2 and Figure 3). These findings demonstrate that balloon injury induces the expression of inflammatory factors, and treatment with SPC can alleviate such effects.

### 3.3. Balloon Injury Decreases the Expression of eNOS, and Its Level Can Be Elevated by SPC

**Figure 4:**
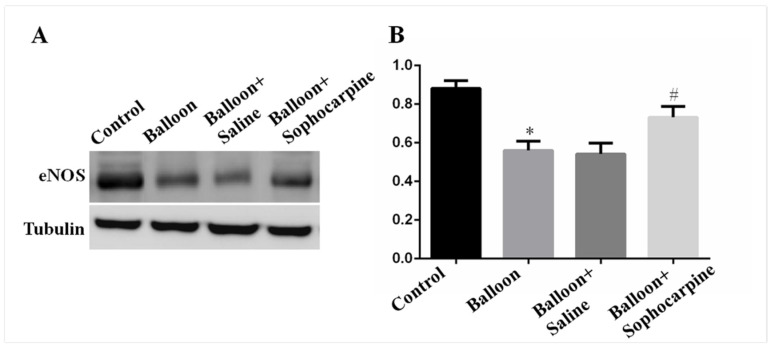
Expression of eNOS in various groups. (**A**) Western blot analysis showed that balloon injury significantly decreased the expression of eNOS, while sophocarpine induced the expression. (**B**) Quantification of the Western blot results. * *p* < 0.01 versus the control group, # *p* < 0.05 versus the balloon group.

eNOS can exert a protective effect against the formation of vascular lesions. The results demonstrated that eNOS level was markedly lower (*p* < 0.01) in balloon injury group than in control group (Figure 4). Interestingly, SPC could increase the protein level of eNOS (*p* < 0.05), as indicated by higher eNOS levels in the rat carotid arteries of balloon injury plus SPC administration group than those of balloon injury group (Figure 4). Our results suggest that SPC plays a protective role in mediating intimal hyperplasia via elevation of eNOS level.

## 4. Discussion

This study found that SPC can attenuate restenosis after angioplasty by reducing the protein levels of inflammatory cytokines and increasing the expression of eNOS., and that showed SPC has potential therapeutic effect on atherosclerotic diseases. Atherosclerosis has been regarded as one of the most important factors that threaten human health. In the initial phase of atherosclerosis, various drugs have been used to prevent the development of cardiovascular diseases, including Simvastatin, Lifibrate, Vitamin E and so on [15]. However, if the vascular lumen has been narrowed or occluded, an invasive operation must be carried out. Although angioplasty is the most commonly used treatment for atherosclerosis-related diseases, the high rate of restenosis is the bottleneck of this therapeutic strategy [4]. Previous studies have demonstrated that the restenosis of vascular lumen after angioplasty is mainly caused by intimal hyperplasia, which can be characterized as smooth muscle cell proliferation, oxidative stress, inflammatory injury, and smooth muscle cell migration [16,17]. To overcome this, we investigated the possible roles of SPC in attenuating intimal hyperplasia and preventing restenosis after angioplasty.

The rat carotid artery balloon injury model was established to imitate the process of balloon angioplasty in clinical settings. Histopathological changes in balloon injury-induced rat carotid arteries were subsequently examined. The results show that balloon injury directly triggers intimal hyperplasia in rat carotid artery, and SPC can ameliorate this effect. These histopathological alterations are consistent with previous clinical observations [18]. Our findings also indicate that SPC may exhibit protective effects on vascular injury after angioplasty.

Given that inflammation is involved throughout the course of atherosclerotic diseases, we speculate that the mechanisms underlying balloon-induced vascular damage and the protective effects of SPC may be related to its anti-inflammatory activity. As expected, SPC could suppress the overexpression of inflammatory cytokines in vascular wall. Previous research has shown that IL-6, IL-1β, MCP-1, NF-κB, TNF-α, ICAM-1 and VCAM-1 are all involved in the process of vascular inflammation [19]. Moreover, the elevated levels of IL-6, IL-1β, NF-κB and TNF-αhave been detected in atherosclerotic plaque [20], and the over-expression of MCP-1, ICAM-1 and VCAM-1 are associated with cigarette smoking-induced inflammatory cell infiltration in rat carotid arteries [18]. NF-κB signaling pathway is the most important marker for inflammation, and it can regulate the expressions of many cytokine-related molecules, such as IL-6, IL-1β, MCP-1, TNF-α, VCAM-1, ICAM-1 and so on [21,22]. Other investigations have also shown that NF-κB is a key player in the inflammatory reaction of cardiovascular diseases [23], and SPC can remarkably suppress NF-κB-mediated inflammation [24]. Our results are in good agreement with the above finding, and thus, it can be concluded that SPC ameliorates vascular inflammation by downregulating NF-κB expression.

Furtherly, the expression of eNOS was determined. As an important protective molecule in the vascular wall, eNOS can protect against artery injuries by preventing the migration and proliferation of vascular smooth muscle cells, promoting the reendothelialization of injured artheries, and inducing the production of nitric oxide [25,26]. A previous study has reported that eNOS alleviates the neointimal formation of rat carotid artery induced by balloon injury [27]. This suggests that SPC may prevent restenosis in balloon-injured carotid artery by increasing eNOS expression levels.

In summary, these findings suggest a protective role of SPC in mediating vascular inflammation triggered by balloon injury, and support the notion that SPC exerts a promising therapeutic potential for curing restenosis in cardiovascular disease patients treated with interventional angioplasty. There are also some limitations in this study. We did not follow ARRIVE guidelines during the study strictly. Further research should be done on the mechanism of SPC alleviating inflammation.

## Data Availability

The data presented in this study are available on request from the corresponding author. The data are not publicly available due to some related studies being carried out.
